# Impact of an Infectious Disease Specialist on an Antimicrobial Stewardship Program at a Resource-Limited, Non-Academic Community Hospital in Korea

**DOI:** 10.3390/jcm8091293

**Published:** 2019-08-23

**Authors:** Yong Chan Kim, Eun Jin Kim, Jung Yeon Heo, Young Hwa Choi, Jin Young Ahn, Su Jin Jeong, Nam Su Ku, Jun Yong Choi, Joon-sup Yeom, Ha Yan Kim

**Affiliations:** 1 Department of Infectious Diseases, Ajou University School of Medicine, Suwon 16499, Korea; 2 Department of Medicine, Yonsei University College of Medicine, Seoul 03722, Korea; 3 Department of Internal Medicine, Yonsei University College of Medicine, Seoul 03722, Korea; 4 Biostatistics Collaboration Unit, Yonsei University College of Medicine, Seoul 03722, Korea

**Keywords:** anti-bacterial agents, antimicrobial stewardship program, infectious disease specialist, antimicrobial resistance, community hospital, Korea

## Abstract

Background: Implementing a successful antimicrobial stewardship program (ASP) is difficult for non-academic community (NAC) hospitals due to insufficient infrastructure. Aim: We evaluated the impact of an infectious disease specialist (IDS) on implementing an ASP in a resource-limited setting in Korea. Methods: A retrospective study was performed at a NAC hospital between June 2015 and August 2018. An IDS has led an ASP at the hospital since June 2017. We used an interrupted time series analysis to evaluate longitudinal effects of the IDS-led ASP on the amount of antibiotic use and incidence of multidrug-resistant organism (MDRO) acquisition. Findings: Total antibiotic use changed from 698.82 ± 74.41 to 602.09 ± 69.94 defined daily dose/1000 patient-days (PDs) after intervention. An immediate reduction in the use of carbapenems, glycopeptides, penicillins, and other antibiotics followed the IDS-led ASP. The 3rd/4th generation cephalosporins and carbapenems prescription rates decreased in slope after the intervention. Incidence of MDRO acquisition changed from 1.38, 0.78, and 0.21/1000 PDs to 1.06, 0.15, and 0.32/1000 PDs in methicillin-resistant *Staphylococcus aureus*, multidrug-resistant *Acinetobacter baumannii*, and multidrug-resistant *Pseudomonas aeruginosa*, respectively. The incidence of methicillin-resistant *Staphylococcus aureus* and multidrug-resistant *Acinetobacter baumannii* acquisition immediately decreased following intervention. Conclusion: An IDS can implement a successful ASP by reducing antibiotic consumption and MDRO acquisition at resource-limited NAC hospitals.

## 1. Introduction

Use of antimicrobial agents has reduced mortality in infectious diseases. However, excessive antibiotic use has caused development of antimicrobial resistance (AMR) in bacteria [1,2]. Infection with an antimicrobial resistant organism has become a major public health concern due to its difficulty to treat, resulting in an increase in hospital stays, cost, and mortality [3]. While antimicrobial resistant organisms have been increasing rapidly, only a few new drugs for combating these pathogens have been developed [4].

An antimicrobial stewardship program (ASP) is one of the most important strategies for preventing AMR [5,6]. In addition, an ASP can improve clinical outcomes in patients while reducing adverse drug reactions [7]. With the proven advantages of ASPs, the necessity for such programs has been realized in most hospitals, regardless of bed size or teaching status. The importance of the role of an infectious disease specialist (IDS) in implementing a successful ASP has been proven through previous studies [8,9,10]. However, most of these studies evaluated the role of an IDS and the effects of an ASP in large, academic hospitals.

On the other hand, only a few studies have reported successful implementation of ASPs in non-academic community (NAC) hospitals [11,12]. NAC hospitals would need modified strategies to implement ASPs because they have fewer resources to allocate to them than do large or teaching hospitals. In Korea, while most NAC hospitals have been challenged to implement ASPs, most of them do not have facility leadership support available. Most IDSs have worked in large, academic hospitals, and they are rarely hired by NAC hospitals.

Since the Middle East respiratory syndrome-coronavirus outbreak in 2015, the Korean government has been encouraging hospitals to employ infection control doctors by policy. Consequently, the Korean National Health Insurance Service now reimburses infection control costs on the condition that doctors and nurses be allocated for infection control in hospitals with more than 300 beds [13]. Accordingly, there has been a recent increase in the number of IDSs working as infection control doctors in NAC hospitals. These IDSs are expected to play a major role in ASPs; however, this has not yet been fully evaluated.

We hypothesized that having an IDS could be a potential solution for implementing a successful ASP in a resource-limited setting. The aim of this study was, therefore, to evaluate the impact of an IDS-led ASP through the changes in the amount of antibiotic use and the rate of multidrug-resistant organism (MDRO) acquisition at a resource-limited NAC hospital in Korea.

## 2. Materials and Methods

### 2.1. Study Population and Data Collection

We conducted a retrospective study at Kimpo Woori Hospital, a NAC hospital with 402 beds in Korea. All patients admitted to the hospital between June 2015 and August 2018 were eligible to participate. Of these, we included 578 patients who had positive cultures for methicillin-resistant *Staphylococcus aureus* (MRSA), multidrug-resistant *Acinetobacter baumannii* (MDRAB), and multidrug-resistant *Pseudomonas aeruginosa* (MDRPA) from any type of specimen received at least 48 h after admission. Multidrug-resistance was defined as resistance to at least one antibiotic between carbapenems, aminoglycosides, and fluoroquinolones.

Data of all patients were included for statistical analysis only once for each organism. We collected the data on clinical characteristics from electronic medical records. Patients’ underlying comorbidities were defined using the International Classification of Diseases, 10th revision. Microorganisms were identified using a VITEK-2 automated bacterial identification system (bioMerieux, Marcy-I’Etoile, France). Antimicrobial susceptibility testing was interpreted using the Clinical and Laboratory Standards Institute guidelines [14]. This study was approved by the Institutional Review Board of Yonsei University Health System Clinical Trial Center (approval number 4-2019-0519), and the protocol adhered to the tenets of the Declaration of Helsinki. Since the study was retrospective in nature and the study participants were anonymized, the Institutional Review Board waived the requirement for written consent from the patients.

### 2.2. Amount of Antibiotic Consumption

We collected the data for monthly antibiotic prescriptions to evaluate the trend in the amount of antibiotic use. This study included only parenteral antibiotics and excluded oral or topical agents. Classification and defined daily doses (DDDs) of antibiotics were determined according to the World Health Organization Anatomical Therapeutic Chemical Classification [15]. We grouped antibiotics as follows: aminoglycosides, 1st/2nd and 3rd/4th generation cephalosporins, carbapenems, glycopeptides, fluoroquinolones, penicillins, and other antibiotics, including linezolid, colistin, and tigecycline.

### 2.3. The Intervention

In March 2015, the hospital implemented an initial ASP led by an internal medicine physician without infectious disease training. Associated polices were defined, and bylaws were introduced throughout the hospital. The policies mainly aimed to limit inappropriate use of prophylactic antibiotics for surgery, which restricted prescription of 3rd generation cephalosporins and aminoglycoside for surgery. Additionally, computerized prescription monitors produced pop-up alarms when physicians continued to prescribe antibiotics designated as restricted, such as linezolid, colistin, tigecycline, glycopeptides, and carbapenems, for more than 3 days. The pop-up alarm system did not restrict or monitor prescription of antibiotics designated as restricted.

The hospital hired an IDS for the first time in March 2017. Three months later, the IDS began to lead the ASP. The ASP was enforced to reduce MDROs and the amount of antibiotic use in the hospital beginning in June 2017. The IDS intended to reduce the use of inappropriate antibiotic combinations, such as overlap in antimicrobial coverage, as well as inappropriate use of prophylactic antibiotics for surgery. Furthermore, he endeavored to increase the appropriate use of carbapenems, glycopeptides, anti-pseudomonal cephalosporins (ceftazidime and cefepime), and other antibiotics (linezolid, colistin, and tigecycline). All physicians were required to consult the IDS for prescription of antibiotics designated as restricted. The IDS assessed the appropriateness of using those antibiotics and recommended the proper dosage and interval and optimal duration of treatment through consultations with the physicians. He also checked the results of culture studies at 5 to 7 days after the approval of antibiotic use and made the decision whether to continue a prescription.

### 2.4. Statistical Analysis

The change in pattern of antibiotic consumption and MDRO acquisition in accordance with the IDS-led ASP between June 2015 and August 2018 was analyzed using an interrupted time series [16]. After implementation of the intervention in June 2017, we set a transition period of three months to allow the intervention process to stabilize. This study divided the time series into two segments, pre- and post-intervention. We used segmented regression analysis to assess the longitudinal effect of the IDS-led ASP. Parameters used in segmented regression analysis were defined as follows. The level was considered the value of a measure at the beginning of a time segment. The trend was considered the rate of change for the measure during the given time interval. The level change after intervention was considered the average value of change after intervention, reflecting the immediate effect of the intervention. The trend change after intervention was considered to be the change in slope during the period after intervention, constituting a gradual change during the segment. The Durbin–Watson statistic was used to test for the presence of autocorrelation, and we verified no serious autocorrelation. We compared the clinical characteristics of each patient with MDRO acquisition for one year before and after the intervention using either the independent t-test or the Chi-square test.

Statistical analyses were performed using SAS version 9.4 (SAS Institute, Inc., Cary, NC, USA.) or R software version 3. Statistical significance was considered as *p* < 0.05.

## 3. Results

### 3.1. Clinical Characteristics of Patients

Between June 2016 and August 2018, the most prevalent organism reported in clinical specimens was MRSA (n = 362), followed by MDRAB (n = 139) and MDRPA (n = 77) (Table 1). Clinical characteristics of patients with hospital-acquired MRSA and MDRPA acquisition did not differ statistically before and after the intervention. Among the patients with MDRAB acquisition, the proportion of those having solid cancer or cardiovascular disease were higher in the post-intervention period than in pre-intervention.

### 3.2. Amount of Antibiotic Consumption

**Figure 1:**
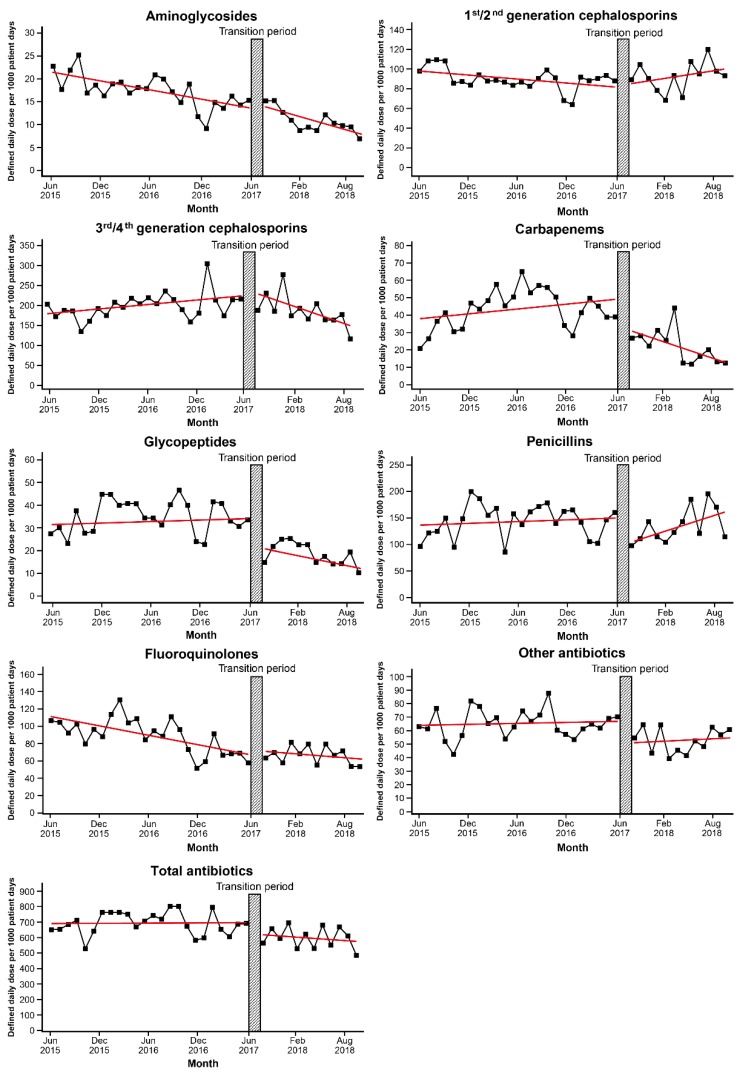
Trends in antibiotic use before and after implementation of the antimicrobial stewardship program led by an infectious disease specialist. Transition period is a three-month lag allowing for the intervention process to stabilize.

Total antibiotic use changed from 698.82 ± 74.41 DDD/1000 patient-days (PDs) to 602.09 ± 69.94 DDD/1000 PDs during study period (Table S1). Following the intervention, amount of most antibiotic use decreased, except 1st/2nd generation cephalosporin, which increased from 93.76 ± 10.13 DDD/1000 PDs to 96.2 ± 14.15 DDD/1000 PDs. We demonstrated antibiotic consumption patterns between June 2015 and August 2018 in Figure 1.

According to segmented regression analysis, there was a decreasing trend in the amount of aminoglycoside and quinolone consumption before the intervention (*p* < 0.001 for each antibiotic) (Table 2). Following the intervention, decreases in both level and trend of the total amount of antibiotics consumed were not statistically significant. However, the intervention had a prominent effect on the use of antibiotics designated as restricted. Just after the transition period, among the antibiotics designated as restricted, there was an immediate reduction in the use of carbapenems (coefficient −16.108, *p* = 0.033), glycopeptides (coefficient −12.287, *p* = 0.012), and other antibiotics (coefficient −16.092, *p* = 0.043). Although significant immediate change was not observed in the amount of 3rd/4th generation cephalosporins use, including that of ceftazidime/cefepime, there was a significant decreasing trend following the intervention as compared with that during pre-intervention (coefficient −8.459, *p* = 0.004). In addition to the immediate response, use of carbapenems showed a significant decreasing trend after the intervention (coefficient −2.029, *p* = 0.027). Penicillins were not subject to restriction, but their use decreased immediately after the intervention. Following an immediate reduction after the transition period, there was no significant change in the trend of use of glycopeptides, penicillins, and other antibiotics.

### 3.3. Incidence of MDRO Acquisition

**Figure 2:**
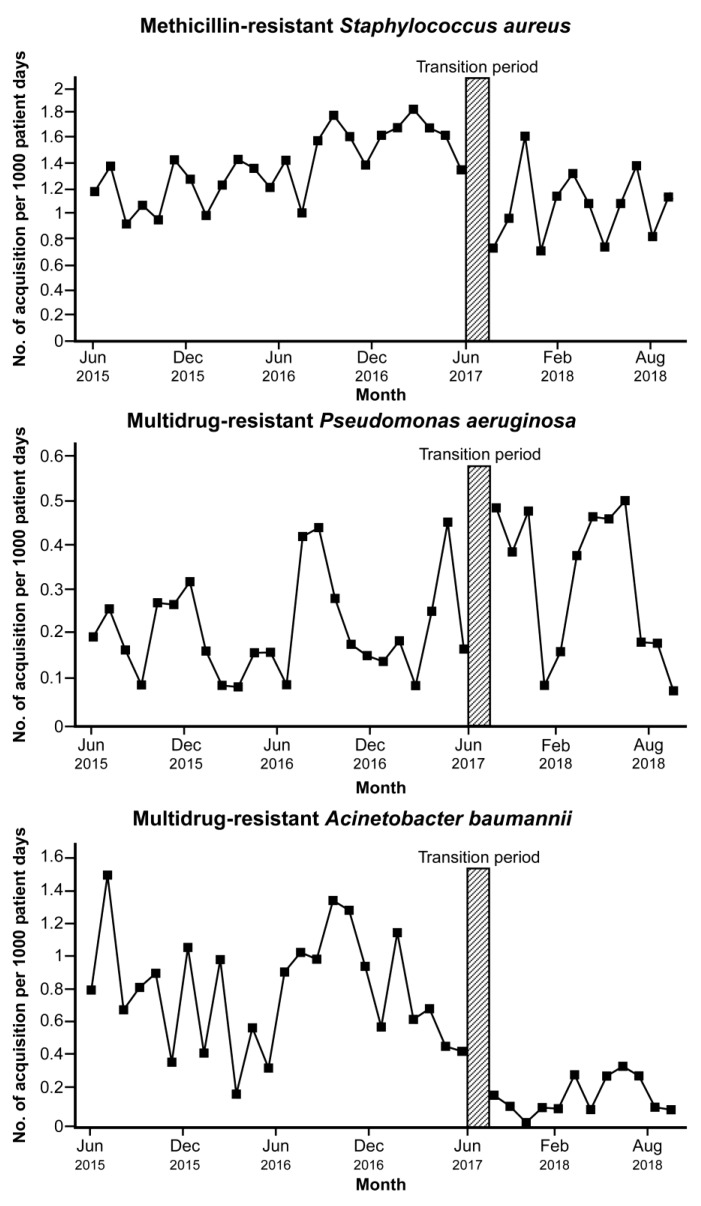
Trends in the incidence of multidrug-resistant organisms acquisition among patients in the hospital before and after implementation of the antimicrobial stewardship program led by an infectious disease specialist. Transition period is a three-month lag allowing for the intervention process to stabilize.

From June 2015 to May 2017 (the period before the intervention), the incidence of MDRO acquisition was 1.381, 0.213, and 0.777/1000 PDs in MRSA, MDRAB, and MDRPA, respectively (Table S2). The incidence of MRSA and MDRAB acquisition decreased following the intervention (1.06/1000 PDs in MRSA and 0.15/1000 PDs in MDRAB). However, during the same period, there was an increase in the incidence of MDRPA acquisition (0.32/1000 PDs). The incidence of MDRO acquisition in patients admitted to the hospital during the study period is presented in Figure 2.

In segmented regression analysis, a significant increasing trend was observed in the incidence of MRSA acquisition before the intervention (coefficient 0.03, *p* < 0.001) (Table 3). Immediately following the transition period, the estimated incidence of MRSA acquisition decreased (coefficient −0.71, *p* < 0.001). There was no significant changing trend in the incidence of MDRAB and MDRPA before the intervention. The incidence of MDRAB acquisition dropped after the transition period (coefficient −0.62, *p* = 0.014); however, MDRPA acquisition increased (coefficient 0.21, *p* = 0.035). There was no significant trend change in the incidence of acquisition of each MDRO after the intervention.

## 4. Discussion

Here, we have demonstrated the impact of an IDS on the implementation of an ASP at a resource-limited NAC hospital in Korea. The intervention had an immediate effect on the reduction in antibiotics designated as restricted. Moreover, there was a continuous decreasing trend in the use of some antibiotics after the intervention. In addition, along with the decreased amount of antibiotic use, the incidence of MRSA and MDRAB acquisition decreased immediately following the IDS-led ASP without a significant trend change after the immediate effect.

Data on antibiotic use in NAC hospitals are limited in Korea. We previously reported on the amount of antibiotic use in a large, university-affiliated hospital [17]. Compared with the rate at this major teaching hospital, our current study demonstrated a similar usage rate of antibiotics in the NAC hospital. Other studies have also reported that hospital size is not important in predicting the amount of antibiotic use [18,19]. Given these findings, regardless of bed size or medical school affiliation, all hospitals should be required to implement ASPs. However, the majority of Korean NAC hospitals still do not have these programs because of insufficient human, financial, and information technology resources as compared with large, academic medical centers. For example, a pharmacist should be involved in an ASP; however, most Korean NAC hospitals do not have a staff member with drug expertise as one of the core elements for a successful hospital ASP [20]. In addition, although NAC hospitals do implement ASPs, there are barriers to the success of these programs. First, a large proportion of patients in NAC hospitals come from long-term care facilities; therefore, they are likely to suffer from recurrent aspiration pneumonia or severe decubitus ulcers, which result in an increased chance of antibiotic use [21]. Second, compared to in large academic hospitals, reducing antibiotic use, especially well-reimbursed drugs, may not be financially beneficial in NAC hospitals [12]. Therefore, ASPs may not be receiving sufficient support from hospital leadership.

Having an IDS could be a potential solution for implementation of a successful ASP at disadvantaged NAC hospitals. IDS consultation can reduce antibiotic use through the discontinuation of inappropriate antibiotics and an increase in appropriate antibiotic use by changing to narrow spectrum antibiotics [22]. In addition, involvement of an IDS in clinical practice has been associated with improved patient outcomes [23]. The skill set of an IDS engenders trust in other physicians, making them receptive to IDS suggestions for appropriate antibiotic use. In this study, the use of 1st/2nd generation cephalosporins tended to increase after the intervention (coefficient 1.891, *p* = 0.061), although these results were not statistically significant. This may have been caused by the change made by the IDS from empirical antibiotics that had been improperly used to appropriate antibiotics.

Similar studies have been conducted in small NAC hospitals with fewer than 200 beds. In these studies, ASPs have also been proven to reduce antibiotic use, lower medical costs, and improve antibiotic susceptibilities to specific pathogens [24,25,26,27]. Even though IDSs spent much less time on the ASP than that in our study, they demonstrated successful results after implementation of ASP. However, key differences exist in our study compared to these other studies. First, most of the hospitals had pharmacists who were dedicated to the ASP and facilitated the intervention, but we did not have a pharmacist as a member of the ASP team because of the lack of available pharmacists in Korean NAC hospitals. Second, it takes less time to develop and maintain an ASP in smaller hospitals. Stenehjem et al. [12] reported that the average time spent on the ASP is 5–10 hours per week in hospitals with 70–150 beds. In contrast, in our study the IDS was required to work full time in the hospital, which has 402 beds, and dedicate the most time to ASP activities. In addition, a low volume of patients and staff members means that once IDS-led ASPs are well established in smaller NAC hospitals, we can expect more rapid effects and better compliance than in larger NAC hospitals. Therefore, we think that this study, conducted in moderate-sized, resource-limited NAC hospital, is worthwhile.

There are multiple factors associated with the development of AMR. Several studies have shown strong evidence suggesting that an effective ASP can reduce the prevalence of AMR, as well as antibiotic use [10,28,29,30]. Our study also shows that an IDS-led ASP can effect a reduction in AMR. We evaluated the incidence of MRSA, MDRAB, and MDRPA acquisition as indicators of AMR. In Korea, these pathogens are not only isolated frequently in clinical specimens, but also cause relevant infections in clinical practice. About 66% of *Staphylococcus aureus* has methicillin resistance, and the carbapenem resistance of *Acinetobacter baumannii* and *Pseudomonas aeruginosa* are 85% and 35%, respectively [31]. However, we were unable to evaluate the incidence of carbapenem-resistant *Enterobacteriaceae* (CRE), because there were few cases of hospital-acquired CRE in the facility studied. Most CRE cases were confirmed in specimens collected prior to 48 h after admission.

A limitation of this study is that an infection control program could be a confounding factor for a decrease in the incidence of MDRO acquisition. However, this factor is unlikely to affect our results. The IDS spent some time meeting with the infection control team—which had been in existence and was doing well in the hospital before he arrived—to implement infection control activities. There was no significant change in the methods of the infection control program. For example, the main strategy for hand hygiene was education, monitoring, and feedback during the study period, and the performance rate was maintained at 81.3% to 88.4%. Another limitation was the immediate increase in the incidence of MDRPA acquisition despite a significant decrease in carbapenem use. A previous study showed that reduced carbapenem use was associated with a decrease in carbapenem resistance in *Pseudomonas aeruginosa* [28], although we did not observe the same results. However, although there was no statistical significance, incidence of MDRPA acquisition had decreased in slope following the intervention.

## 5. Conclusions

ASP is an effective strategy to prevent or slow the development of MDROs. However, it is difficult for NAC hospitals to implement a successful ASP due to the lack of multidisciplinary resources. We have shown that an IDS could be a potential solution for successful implementation of an ASP in resource-limited settings. Our findings should, however, be verified in different settings, such as smaller institutions, thus facilitating the need for further study.

## Figures and Tables

**Table 1:** Clinical characteristics of patients with hospital-acquired multidrug-resistant organisms between June 2016 and August 2018.

Variables	Methicillin-Resistant *Staphylococcus aureus*			Multidrug-Resistant *Pseudomonas aeruginosa*			Multidrug-Resistant *Acinetobacter baumannii*		
	One Year before Intervention (*n* = 217)	One Year after Intervention (*n* = 145)	*p*-value	One Year before Intervention (*n* = 33)	One Year after Intervention (*n* = 44)	*p*-value	One Year before Intervention (*n* = 118)	One Year after Intervention (*n* = 21)	*p*-value
Age, years (IQR)	77 ± 13.6	75 ± 15.98	0.184	77 ± 13.33	73 ± 15.13	0.26	76 ± 14.26	77 ± 11.34	0.73
Male, *n* (%)	129 (59.5)	97 (66.9)	0.152	16 (48.5)	25 (56.8)	0.468	38 (32.2)	7 (33.3)	0.919
Antibiotic use in the past 30 days, *n* (%)	166 (76.5)	95 (65.5)	0.065	27 (81.8)	40 (90.9)	0.291	107 (90.7)	18 (85.7)	0.536
Comorbidities, *n* (%)									
Diabetes	73 (33.6)	41 (28.3)	0.282	13 (39.4)	10 (22.7)	0.114	37 (31.4)	8 (38.1)	0.543
Solid cancer	34 (15.7)	28 (19.3)	0.278	6 (18.2)	7 (15.9)	0.792	21 (17.8)	8 (38.1)	0.044
Hematologic malignancy	1 (0.5)	2 (1.4)	0.567	1 (3.0)	1 (2.3)	>0.999	1 (0.9)	0 (0.0)	>0.999
Solid organ transplantation	0 (0.0)	0 (0.0)	-	0 (0.0)	0 (0.0)	-	0 (0.0)	0 (0.0)	-
Hematologic stem cell transplantation	0 (0.0)	1 (0.7)	0.401	0 (0.0)	1 (2.3)	>0.999	0 (0.0)	0 (0.0)	-
Rheumatologic disease	4 (1.8)	2 (1.4)	>0.999	0 (0.0)	0 (0.0)	-	0 (0.0)	0 (0.0)	-
Cardiovascular disease	139 (64.1)	96 (66.2)	0.674	20 (60.6)	28 (63.6)	0.786	73 (61.9)	18 (85.7)	0.034
Chronic obstructive pulmonary disease	33 (15.2)	26 (17.9)	0.492	2 (6.1)	8 (18.2)	0.174	18 (15.3)	5 (23.8)	0.344
Renal Disease	60 (27.7)	42 (29.0)	0.785	6 (18.2)	7 (15.9)	0.792	30 (25.4)	6 (28.6)	0.762
Liver disease	45 (20.7)	32 (22.1)	0.762	4 (12.1)	9 (20.5)	0.334	18 (15.3)	3 (14.3)	>0.999
HIV infection	0 (0.0)	0 (0.0)	-	0 (0.0)	0 (0.0)	-	0 (0.0)	0 (0.0)	-
Hemodialysis	16 (7.4)	10 (6.9)	0.863	2 (6.1)	6 (13.6)	0.454	8 (6.8)	2 (9.5)	0.648
Neutropenia	0 (0.0)	1 (0.7)	0.401	0 (0.0)	0 (0.0)	-	0 (0.0)	0 (0.0)	-
Steroid therapy	16 (7.4)	10 (6.9)	>0.999	2 (6.1)	2 (4.6)	0.347	14 (11.9)	2 (9.5)	>0.999
Immunosuppressive therapy	0 (0.0)	0 (0.0)	-	0 (0.0)	0 (0.0)	-	0 (0.0)	0 (0.0)	-
Chemotherapy	0 (0.0)	3 (2.1)	0.064	0 (0.0)	1 (2.3)	>0.999	0 (0.0)	0 (0.0)	-
Radiotherapy	0 (0.0)	0 (0.0)	-	0 (0.0)	0 (0.0)	-	0 (0.0)	0 (0.0)	-

Note: IQR, interquartile range; HIV, human immunodeficiency virus.

**Table 2:** Segmented regression model predicting monthly daily defined dose per 1000 patient-days in the hospital.

Antibiotics	Coefficient	Standard Error	*p*-Value
Aminoglycosides			
Baseline level	22.0256	0.9929	
Baseline trend	−0.3305	0.0695	<0.001
Level change after intervention	0.8123	1.7243	0.641
Trend change after intervention	−0.2064	0.2089	0.331
1st/2nd generation cephalosporins			
Baseline level	101.9253	4.6246	
Baseline trend	−0.6535	0.3237	0.052
Level change after intervention	1.9191	8.0315	0.813
Trend change after intervention	1.891	0.9732	0.061
3rd/4th generation cephalosporins			
Baseline level	177.211	13.0193	
Baseline trend	1.7935	0.9112	0.058
Level change after intervention	11.108	22.6103	0.627
Trend change after intervention	−8.4598	2.7399	0.004
Ceftazidime/cefepime			
Baseline level	66.3764	8.7921	
Baseline trend	0.5003	0.6153	0.422
Level change after intervention	−7.0211	15.269	0.649
Trend change after intervention	−5.663	1.8503	0.004
Carbapenems			
Baseline level	39.3779	4.1486	
Baseline trend	0.4704	0.2903	0.115
Level change after intervention	−16.1077	7.2047	0.033
Trend change after intervention	−2.0287	0.8731	0.027
Glycopeptides			
Baseline level	35.0324	2.6622	
Baseline trend	0.1151	0.1863	0.541
Level change after intervention	−12.2869	4.6233	0.012
Trend change after intervention	−0.885	0.5602	0.124
Penicillins			
Baseline level	140.8157	12.7416	
Baseline trend	0.5614	0.8917	0.533
Level change after intervention	−46.7517	22.128	0.043
Trend change after intervention	4.2492	2.6814	0.123
Fluoroquinolones			
Baseline level	115.0862	6.0155	
Baseline trend	−1.8856	0.421	<0.001
Level change after intervention	4.1865	10.447	0.691
Trend change after intervention	1.0813	1.2659	0.399
Other antibiotics			
Baseline level	64.8456	4.395	
Baseline trend	0.1295	0.3076	0.677
Level change after intervention	−16.0924	7.6328	0.043
Trend change after intervention	0.185	0.9249	0.843
Total antibiotics			
Baseline level	696.3197	31.5014	
Baseline trend	0.2003	2.2046	0.928
Level change after intervention	−73.2129	54.7076	0.19
Trend change after intervention	−4.1733	6.6293	0.536

**Table 3:** The incidence of multidrug-resistant organisms acquisition among patients in the hospital over time using segmented regression analysis.

Multidrug-Resistant Organisms	Coefficient	Standard Error	*p*-Value
Methicillin-resistant *Staphylococcus aureus*			
Baseline level	1.05	0.10	
Baseline trend	0.03	0.01	0.001
Level change after intervention	−0.71	0.18	<0.001
Trend change after intervention	−0.02	0.02	0.439
Multidrug-resistant *Pseudomonas aeruginosa*			
Baseline level	0.19	0.06	
Baseline trend	0.002	0.004	0.628
Level change after intervention	0.21	0.10	0.035
Trend change after intervention	−0.02	0.01	0.082
Multidrug-resistant *Acinetobacter baumannii*			
Baseline level	0.85	0.13	
Baseline trend	−0.01	0.01	0.569
Level change after intervention	−0.62	0.24	0.014
Trend change after intervention	0.02	0.03	0.588

## Supplementary Materials

https://www.mdpi.com/2077-0383/8/9/1293/s1. Table S1: Amount of antibiotic consumption before and after the intervention. Table S2: The incidence of multidrug-resistant organisms before and after intervention.

## References

[1] Ventola C.L. (2015). The antibiotic resistance crisis: Part 1: Causes and threats. PT.

[2] Laxminarayan R., Matsoso P., Pant S., Brower C., Rottingen J.A., Klugman K., Davies S. (2016). Access to effective antimicrobials: A worldwide challenge. Lancet.

[3] Golkar Z., Bagasra O., Pace D.G. (2014). Bacteriophage therapy: a potential solution for the antibiotic resistance crisis. J. Infect. Dev. Ctries..

[4] Infectious Diseases Society of America (2010). The 10 x ‘20 Initiative: Pursuing a global commitment to develop 10 new antibacterial drugs by 2020. Clin. Infect. Dis..

[5] Barlam T.F., Cosgrove S.E., Abbo L.M., MacDougall C., Schuetz A.N., Septimus E.J., Srinivasan A., Dellit T.H., Falck-Ytter Y.T., Fishman N.O. (2016). Implementing an antibiotic stewardship program: Guidelines by the Infectious Diseases Society of America and the Society for Healthcare Epidemiology of America. Clin. Infect. Dis..

[6] Baur D., Gladstone B.P., Burkert F., Carrara E., Foschi F., Dobele S., Tacconelli E. (2017). Effect of antibiotic stewardship on the incidence of infection and colonisation with antibiotic-resistant bacteria and Clostridium difficile infection: A systematic review and meta-analysis. Lancet Infect. Dis..

[7] Schuts E.C., Hulscher M., Mouton J.W., Verduin C.M., Stuart J., Overdiek H., van der Linden P.D., Natsch S., Hertogh C., Wolfs T.F.W. (2016). Current evidence on hospital antimicrobial stewardship objectives: A systematic review and meta-analysis. Lancet Infect. Dis..

[8] McQuillen D.P., Petrak R.M., Wasserman R.B., Nahass R.G., Scull J.A., Martinelli L.P. (2008). The value of infectious diseases specialists: non-patient care activities. Clin. Infect. Dis..

[9] Nilholm H., Holmstrand L., Ahl J., Mansson F., Odenholt I., Tham J., Melander E., Resman F. (2015). An audit-based, infectious disease specialist-guided antimicrobial stewardship program profoundly reduced antibiotic use without negatively affecting patient outcomes. Open Forum Infect. Dis..

[10] Hwang H., Kim B. (2018). Impact of an infectious diseases specialist-led antimicrobial stewardship programmes on antibiotic use and antimicrobial resistance in a large Korean hospital. Sci. Rep..

[11] Pollack L.A., van Santen K.L., Weiner L.M., Dudeck M.A., Edwards J.R., Srinivasan A. (2016). Antibiotic stewardship programs in U.S. acute care hospitals: findings from the 2014 National Healthcare Safety Network Annual Hospital Survey. Clin. Infect. Dis..

[12] Stenehjem E., Hyun D.Y., Septimus E., Yu K.C., Meyer M., Raj D., Srinivasan A. (2017). Antibiotic stewardship in small hospitals: barriers and potential solutions. Clin. Infect. Dis..

[13] Kwon K.T., Lee W.K., Yu M.H., Park H.J., Lee K.H., Chae H.J. (2018). The impact of infection control cost reimbursement policy on trends in central line-associated bloodstream infections. Open Forum Infect. Dis..

[14] Clinical and Laboratory Standards Institute Performance standards for antimicrobial susceptibility testing. 27th ed. CLSI supplement M100. https://www.researchgate.net/profile/Nikolaos_Andritsos/post/Where_to_find_MICs_of_antimicrobial_agents/attachment/59d659e279197b80779af319/AS%3A544238144323584%401506767893696/download/2017_CLSI_M100_Performance+Standards+for+Antimicrobial+Susceptibility+Testing_27th+ed..pdf.

[15] WHO Collaborating Centre for Drug Statistics Methodology ATC/DDD index 2019. https://www.whocc.no/atc_ddd_index/.

[16] Wagner A.K., Soumerai S.B., Zhang F., Ross-Degnan D. (2002). Segmented regression analysis of interrupted time series studies in medication use research. J. Clin. Pharm. Ther..

[17] Kim Y.C., Kim M.H., Song J.E., Ahn J.Y., Oh D.H., Kweon O.M., Lee D., Kim S.B., Kim H.W., Jeong S.J. (2013). Trend of methicillin-resistant *Staphylococcus aureus* (MRSA) bacteremia in an institution with a high rate of MRSA after the reinforcement of antibiotic stewardship and hand hygiene. Am. J. Infect. Control.

[18] Couderc C., Lacave L., L’Heriteau F., Astagneau P. (2011). Surveillance of overall hospital antibiotic consumption: is stratification according to hospital size the best method?. Infect. Control Hosp. Epidemiol..

[19] Baggs J., Fridkin S.K., Pollack L.A., Srinivasan A., Jernigan J.A. (2016). Estimating national trends in inpatient antibiotic use among US hospitals from 2006 to 2012. JAMA Intern. Med..

[20] Pollack L.A., Srinivasan A. (2014). Core elements of hospital antibiotic stewardship programs from the Centers for Disease Control and Prevention. Clin. Infect. Dis..

[21] Ohl C.A., Dodds Ashley E.S. (2011). Antimicrobial stewardship programs in community hospitals: The evidence base and case studies. Clin. Infect. Dis..

[22] Ozkurt Z., Erol S., Kadanali A., Ertek M., Ozden K., Tasyaran M.A. (2005). Changes in antibiotic use, cost and consumption after an antibiotic restriction policy applied by infectious disease specialists. Jpn. J. Infect. Dis..

[23] Schmitt S., McQuillen D.P., Nahass R., Martinelli L., Rubin M., Schwebke K., Petrak R., Ritter J.T., Chansolme D., Slama T. (2014). Infectious diseases specialty intervention is associated with decreased mortality and lower healthcare costs. Clin. Infect. Dis..

[24] LaRocco A. (2003). Concurrent antibiotic review programs—A role for infectious diseases specialists at small community hospitals. Clin. Infect. Dis..

[25] Storey D.F., Pate P.G., Nguyen A.T., Chang F. (2012). Implementation of an antimicrobial stewardship program on the medical-surgical service of a 100-bed community hospital. Antimicrob. Resist. Infect. Control.

[26] Day S.R., Smith D., Harris K., Cox H.L., Mathers A.J. (2015). An Infectious Diseases Physician-Led Antimicrobial Stewardship Program at a Small Community Hospital Associated With Improved Susceptibility Patterns and Cost-Savings After the First Year. Open Forum Infect. Dis..

[27] Bartlett J.M., Siola P.L. (2014). Implementation and first-year results of an antimicrobial stewardship program at a community hospital. Am. J. Health Syst. Pharm..

[28] Pakyz A.L., Oinonen M., Polk R.E. (2009). Relationship of carbapenem restriction in 22 university teaching hospitals to carbapenem use and carbapenem-resistant *Pseudomonas aeruginosa*. Antimicrob. Agents Chemother..

[29] Lemmen S.W., Hafner H., Kotterik S., Lutticken R., Topper R. (2000). Influence of an infectious disease service on antibiotic prescription behavior and selection of multiresistant pathogens. Infection.

[30] de Man P., Verhoeven B.A., Verbrugh H.A., Vos M.C., van den Anker J.N. (2000). An antibiotic policy to prevent emergence of resistant bacilli. Lancet.

[31] Kim D., Ahn J.Y., Lee C.H., Jang S.J., Lee H., Yong D., Jeong S.H., Lee K. (2017). Increasing resistance to extended-spectrum cephalosporins, fluoroquinolone, and carbapenem in gram-negative bacilli and the emergence of carbapenem non-susceptibility in *Klebsiella pneumoniae*: Analysis of Korean Antimicrobial Resistance Monitoring System (KARMS) data from 2013 to 2015. Ann. Lab. Med..

