# Supplementary material for: Impact of an Infectious Disease Specialist on an Antimicrobial Stewardship Program at a Resource-Limited, Non-Academic Community Hospital in Korea
Source: J Clin Med. 2019 Aug 23;8(9):1293. doi: 10.3390/jcm8091293 (PMC6780603; doi:10.3390/jcm8091293)
Supplement: Supplementary file 1 [file jcm-08-01293-s001.pdf]

Table S1. Amount of antibiotic consumption before and after the intervention.

Antibiotics	Monthly mean defined daily dose per 1000 patient-days (±standard deviation)	
	**Pre-intervention**	**Post-intervention**
Aminoglycosides	17.895 ± 3.451	11.417 ± 2.533
1st/2nd generation cephalosporins	93.756 ± 10.132	96.204 ± 14.155
3rd/4th generation cephalosporins	199.629 ± 32.13	188.031 ± 39.153
Carbapenems	45.257 ± 10.835	24.43 ± 9.552
Glycopeptides	36.471 ± 6.991	20.503 ± 4.8
Penicillins	147.834 ± 30.459	138.808 ± 32.469
Fluoroquinolones	91.516 ± 20.365	68.79 ± 10.29
Other antibiotics	66.464 ± 10.544	53.906 ± 9.333
Total antibiotics	698.823 ± 74.413	602.088 ± 69.941

Table S2. The incidence of multidrug-resistant organisms before and after intervention.

Multidrug-resistant organisms	Pre-intervention		Post-intervention	
	Case	Incidence	Case	Incidence [1]
Methicillin-resistant *Staphylococcus aureus*	389	1.381	146	1.063
Multidrug-resistant *Acinetobacter baumannii*	60	0.213	44	0.32
Multidrug-resistant *Pseudomonas aeruginosa*	219	0.777	21	0.153

[1] Incidence is the number of cases per 1000 patient-years.
